# Automatic specific absorption rate (SAR) prediction for hyperthermia treatment planning using deep learning method

**DOI:** 10.1080/02656736.2025.2554860

**Published:** 2025-09-09

**Authors:** Yankun Lang, Dario B. Rodrigues, Lei Ren

**Affiliations:** Department of Radiation Oncology Physics, University of Maryland, Baltimore, MD, USA

**Keywords:** Hyperthermia therapy, brain cancer, specific absorption rate, treatment planning, supervised deep learning

## Abstract

**Objective:**

To develop a deep learning method for fast and accurate prediction of Specific Absorption Rate (SAR) distributions in the human head to support real-time hyperthermia treatment planning (HTP) of brain cancer patients.

**Approach:**

We propose an encoder-decoder neural network with cross-attention blocks to predict SAR maps from brain electrical properties, tumor 3D isocenter coordinates and microwave antenna phase settings. A dataset of 201 simulations was generated using finite-element modeling by varying tissue properties, tumor positions, and antenna phases within a human head model equipped with a three-ring phased-array applicator. The model was trained and evaluated on this dataset using standard error metrics and structural similarity analysis.

**Main results:**

On a held-out test set of 20 samples, the model achieved a mean root-mean-squared error (RMSE) of 3.3 W/kg and a mean absolute error (MAE) of 1.6 W/kg across the whole brain. In target regions, RMSE and MAE were 4.8 and 2.5 W/kg, respectively. The structural similarity index (SSIM) reached a mean value of 0.90, and the computation time was reduced from 10 min (simulation-based) to 4 s using our deep learning approach. The proposed method enables accurate, efficient SAR prediction for HTP in the brain, potentially supporting real-time HTP to optimize tumor temperature and improve clinical outcomes.

**Significance:**

This work introduces a novel deep learning-based approach that significantly accelerates SAR calculation in HTP, enabling adaptive therapy strategies to improve treatment outcomes in hyperthermia.

## 1. Introduction

Hyperthermia therapy (HT) is a potent adjuvant treatment used to enhance the efficacy of conventional cancer therapies, including radiation therapy and chemotherapy [1–3]. By delivering controlled and targeted therapeutic heat in the range of 40–44°C for one hour, HT increases the sensitivity of malignant cells to cytotoxic agents, thereby augmenting the therapeutic impact of radiation and chemotherapeutic interventions [4–8]. This synergistic approach aims to improve tumor control and potentially reduce therapeutic doses of radiation and chemotherapeutic agents, thereby minimizing side effects and improving patient outcomes. Hyperthermia is usually added to radiation therapy, chemotherapy, or a combination of both, and more recently has been explored in conjunction with immunotherapy [9–13]. Notably, clinical studies have demonstrated significantly improved survival outcomes rates across a broad spectrum of tumor indications, including breast cancer [14], melanoma [15], glioblastoma [16], head and neck tumors [17,18], cervical cancer [19] and other advanced pelvic tumors [20].

Three-dimensional (3D) hyperthermia treatment planning (HTP) has significantly evolved in recent years and is now a powerful tool to optimize patient-specific treatment quality [21–23], but used routinely only in a few academic medical centers. During HTP, a 3D patient virtual model is created from CT or MR scans and integrated with the HT applicator virtual model to establish the treatment setup in silico. The optimization is achieved via two key parameters: specific absorption rate (SAR) and temperature [24,25]. The SAR parameter is measured in watts per kilogram (W/kg) units and quantifies the rate at which tissues absorb energy from electromagnetic fields induced by radiofrequency or microwave antennas (RF/MW) [26]. Although this parameter does not consider any heat transfer phenomena, tissue electrical properties used in SAR calculations are well characterized with adequate clinical accuracy [24,27]. In contrast, HTP performed via temperature simulations considers SAR, heat transfer in living tissues (also known as bioheat transfer) and important physiological changes such as temperature-dependent blood perfusion [28]. There are significant challenges with bioheat transfer simulations, including high uncertainly associated with blood perfusion properties and significant simplifications in the bioheat transfer models that do not capture complex human physiology and thermoregulation [25]. Still, temperature simulations can provide insights into the treatment such as potential hotspot locations. Directly or indirectly, all HTP strategies for RF/MW phased-array devices use SAR, which takes as inputs brain tissue properties, patient anatomy, and target location to calculate the amplitude and phase settings of the antennas to generate an optimized SAR pattern. This optimization aims to maximize average SAR within the hyperthermia target volume while minimizing SAR in healthy tissues [29].

Current SAR calculations for HTP are based on complex models that simulate electromagnetic field interactions with biological tissues. Many software platforms such as COMSOL Multiphysics (COMSOL, Palo Alto, CA) employ numerical solvers based on the finite element method (FEM) to compute SAR distributions by combining tissue properties - namely, relative permittivity, electrical conductivity and density-with the normalized electric field. The numerical model solves the vector-Helmholtz equation everywhere in the domain for a given frequency to calculate the electric field through wave propagation. However, as opposed to finite difference (FD) based algorithms FEM-based numerical algorithms are computationally demanding and time-consuming, especially when applied to complex, 3D patient-specific models. Consequently, when using general-purpose FEM-based tools like COMSOL, the iterative process of adjusting antenna phase and amplitude settings to optimize therapeutic heating while ensuring safety is too slow to support real-time or adaptive HTP.

Deep learning-based approaches hold significant potential to overcome the computational limitations of FEM-based SAR simulations. In recent years, deep learning-based methods have been increasingly developed by various groups, including ours, for image reconstruction and enhancement to improve the efficiency and accuracy of dose verification in several medical applications [30–34]. For instance, Schwab et al. [30] introduced the DALnet framework, which combines universal backprojection (UBP) with dynamic aperture length (DAL) correction and a deep convolutional neural network (CNN). This approach enables high-resolution, real-time projection imaging of 3D structures at over 50 frames per second and outperforms traditional iterative algorithms in both speed and accuracy. Similarly, Antholzer et al. [31] utilized deep learning to address under-sampling artifacts, replacing time-consuming iterative reconstruction methods with direct algorithms. Jiang et al. [32] employed a 3D U-Net to derive a 3D dose map for dose verification from an enhanced initial pressure map. Lang et al. [33] introduced a hybrid supervised deep learning method for improved reconstruction quality and efficiency. Furthermore, Lang et al. applied their method in patient-specific studies to achieve even greater accuracy in dose verification [34].

However, while these deep learning methods have been primarily developed for 2D or 3D image-based inputs, SAR distribution is fundamentally computed on 3D geometric meshes or point clouds [25]. This distinction requires the use of deep learning techniques designed specifically for 3D geometric data. In recent years, deep learning methods have been developed to process 3D meshes and point clouds [35–39]. While these methods have demonstrated significant advancements in 3D geometric mesh and point cloud analysis, they are not directly applicable to SAR prediction. A key limitation is that existing deep learning methods are designed to process only a single type of input—either meshes or point clouds—whereas SAR prediction requires integrating additional parameters, such as antenna phases and amplitudes. These antenna parameters, which have different dimensions compared to the 3D geometric model, play a crucial role in accurately modeling and predicting SAR distributions. Therefore, adapting deep learning techniques for SAR distribution requires novel architectures capable of jointly processing geometric meshes and structured antenna information. To address this gap, the cross-attention transformer [40], a mechanism commonly used in transformer-based architectures, enables the model to integrate information from one source while using another as contextual input. In the case of SAR prediction for HTP for phased-array systems, cross-attention allows the model to focus on the relevant features of the antenna settings while simultaneously considering the anatomical characteristics of human tissue. This dynamic fusion of data significantly enhances the model’s ability to accurately predict SAR distributions in tissue by accounting for both anatomical and technical parameters, thus improving the precision and reliability of SAR-based treatment planning.

In this study, we propose a deep learning-based model that integrates antenna parameters with patient-specific anatomical data using a cross-attention transformer architecture for SAR prediction. The model is applied to the brain HT applicator described by Rodrigues et al. [26], which consists of a 72-antenna MW phased-array system operating at 915 MHz, with the goal of significantly improving the computational efficiency of HTP.

## 2. Methods

### 2.1. Overview

**Figure 1.**
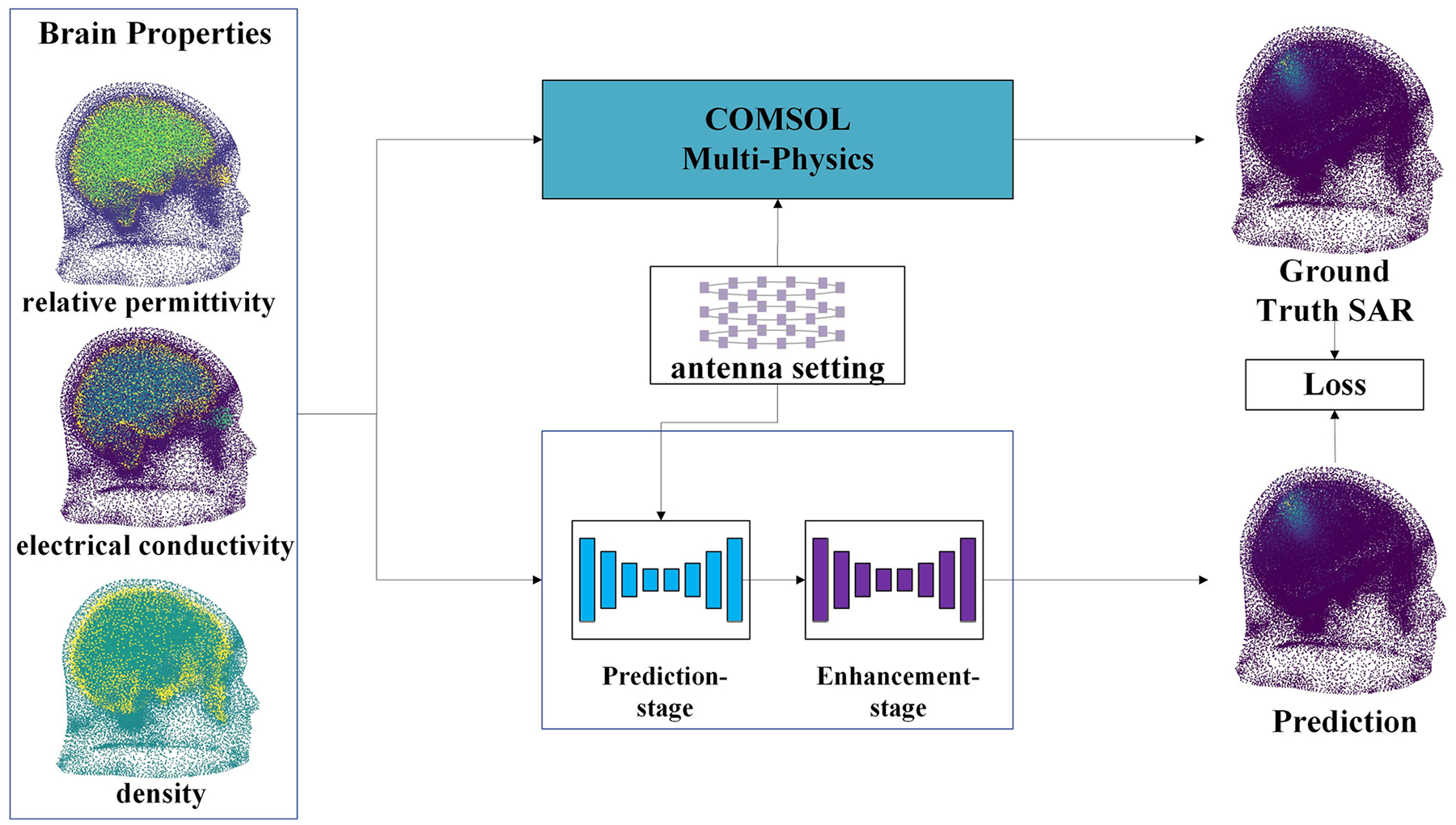
The workflow of our approach for SAR simulation and prediction with a prediction-enhancement strategy.

Figure 1 summarizes the deep learning model proposed in this work, which employs a Prediction-Enhancement two-stage strategy to harness the power of deep learning for both prediction and enhancement of SAR distributions. Specifically, in the prediction-stage, the proposed network directly predicts the SAR distribution by taking the 3D anatomical model and the antenna settings as inputs. The proposed network has an encoder-decoder architecture, in which the encoder consists of four sequential blocks built using cross-attention module. In the enhancement-stage, a 3D U-net is applied to further improve the prediction results. By using the cross-attention module, the network can better explore the relationships between brain electrical properties/anatomy and the antenna parameters.

### 2.2. Data generation

**Figure 2.**
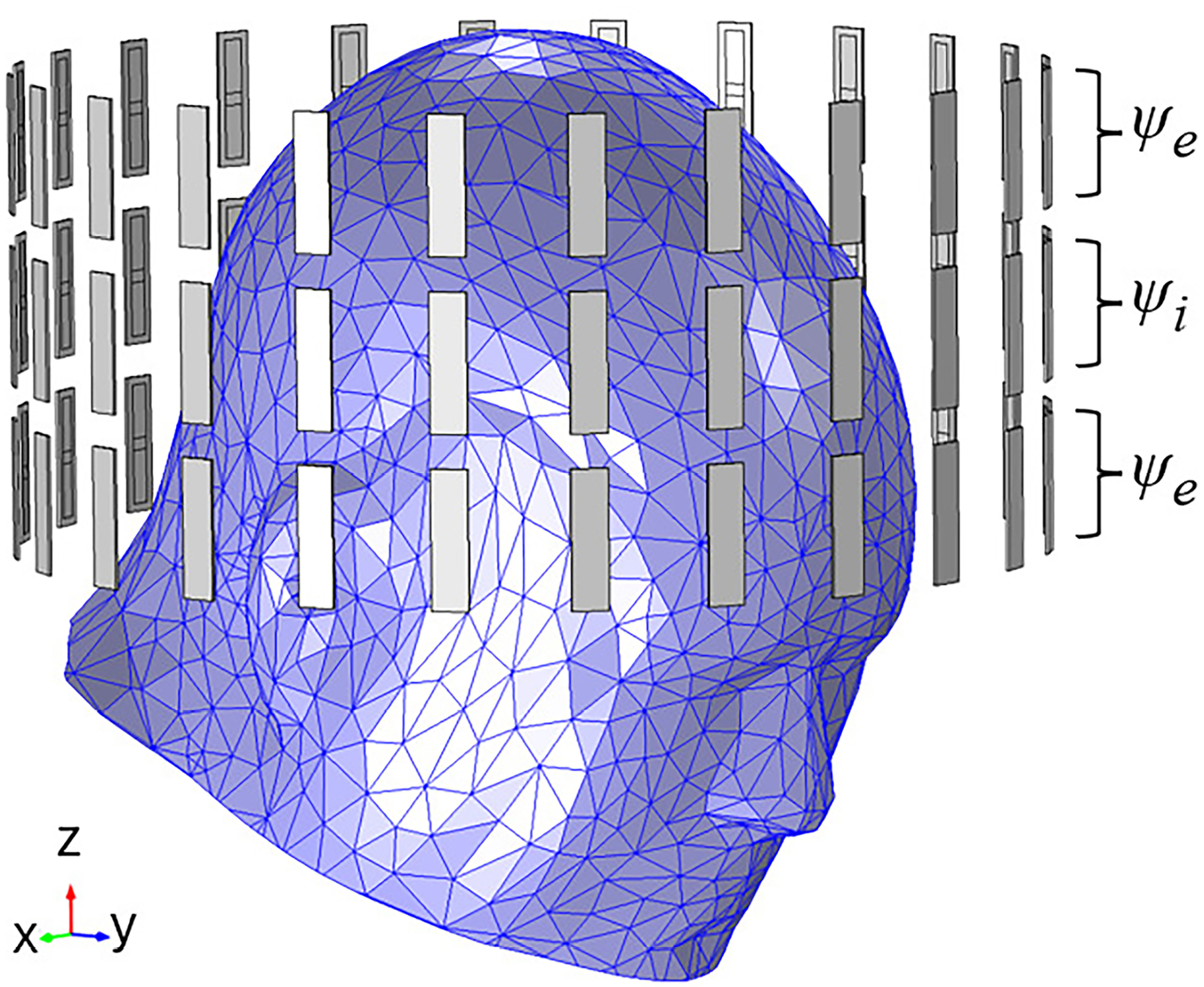
Antenna configuration of the phased-array brain applicator comprised of three rings, with each ring housing 24 antennas. The two external rings shares the same phases, leading to a total of 48 unique phases.

An overview of SAR prediction using a deep learning method is illustrated in Figure 1. The SAR numerical simulations will be computed in the FEM software COMSOL Multiphysics V6.0, serving as the ground truth, that is, the reference standard against which predictions are evaluated. The HT brain applicator case study is illustrated on Figure 2 and consists of 3 rings of 24 dipole antennas enclosed in a cylindrical frame with 26 cm diameter and 13 cm in length [41]. Each dipole consists of a planar bow-tie antenna 30×6 mm^2^ with a 5-mm gap and a minimum width of 2 mm at the feeding port. Although there are 72 antennas, the phase of the antennas in the outer rings $\left(\psi_{e}\right)$ along the z axis is the same, yielding a total of 48 distinct antenna phases. The applicator surrounds a human head model consisting of a simplified truncated version of the VHP-Female model v2.2 (NEVA BioElectromagnetics, USA) with five layers: fat, bone, muscle, brain tissue, and intranasal air. The brain tissue corresponds to the intracranial space, with different properties being analyzed: grey matter, white matter, and average of these brain tissues. Table 1 summarizes these tissues and their electrical properties. Finally, a compartment filled with deionized water is used to couple the energy between the antennas and the human head [42].

The SAR parameter is calculated from the electrical field calculated in COMSOL to solve the Maxwell equations [43] as follows:

(1) $$E_{\text{SAR}}=\frac{\sigma \left|E^{2}\right|}{2\rho }$$

where σ is the electrical conductivity and ρ is the density. The phase (ψ) for each antenna is calculated using the following formulas that are based on the path difference method [26]:

(2) $$\theta_{n}=2(n-1)\frac{\pi }{N},\;n=1,\ldots ,24$$

(3) $$R_{\text{in}}=\sqrt{{\left(a\text{cos}\left(\theta_{n}\right)-x_{0}\right)}^{2}+{\left(a\text{sin}\left(\theta_{n}\right)-y_{0}\right)}^{2}}$$

(4) $$R_{\text{en}}=\sqrt{{\left(a\text{cos}\left(\theta_{n}\right)-x_{0}\right)}^{2}+{\left(a\text{sin}\left(\theta_{n}\right)-y_{0}\right)}^{2}+g^{2}}$$

(5) $$\psi_{\text{in}}=\frac{2\pi }{\lambda_{\text{brain}}}\left(R_{\text{in}}-\text{min}\left(R_{\text{in}}\right)\right)$$

(6) $$\psi_{\text{en}}=\frac{2\pi }{\lambda_{\text{brain}}}\left(R_{\text{en}}-\text{min}\left(R_{\text{in}}\right)\right)$$

where θ is the angle of the antenna relative to the axial plane (referential XY in Figure 2), n in the antenna index in the 24-antenna ring, a is the applicator radius (13 cm), g is the space between rings (4.2 cm), $R_{\text{in}}\left(R_{\text{en}}\right)$ is the distance from the antennas in the central (external) ring to the steering focus ($x_{0},y_{0}$), and $\lambda_{\text{brain}}$ is the wavelength in brain tissue (Table 1). Further numerical methodology and its validation can be found in Rodrigues et al. [26]. The MRI scan used to generate the human model comprised 109 slices with 256×256 voxels each [44]. To match the mesh-element density within the head, the volumetric data was downsampled to 55×50×50 interpolation points, resulting in approximately 100K vertices in the head meshes. The numerical mesh for the human head model consisted of 100,000 elements.

A total of 201 simulation datasets (or samples) were generated for SAR normalized to 100 W, incorporating variations in tissue properties (Table 1) and target locations defined by the steering focus ($x_{0},y_{0}$), thus providing diverse scenarios to train and validate the deep learning model effectively. For each simulation, the target location varied in 2 cm increments within the range [−4 cm,6 cm] along each axis in the axial plane XY, with the starting z-coordinate (z=0cm) aligned with the brain isocenter. Additional samples were simulated in the same axial locations, but for z=−2cm and z=+2cm. We performed a 5 fold cross-validation, with the data split into 160 samples for training, 21 for validation, and 20 for testing. All simulations in this study were generated using a single anatomical head model.

### 2.3. Functional goal

To formalize the SAR prediction task, we define the mapping learned by our model as:

(7) $$\hat{\text{SAR}}=ℱ\left(ℬ,\psi \right),$$

where: $\hat{\text{SAR}}\in R^{N}$ is the predicted specific absorption rate distribution over N mesh points in the brain, $ℬ\in R^{N\times 6}$ represents the spatial and electrical properties of the brain mesh, including 3D coordinates, relative permittivity $\left(\varepsilon_{r}\right)$, electrical conductivity (σ), and density (ρ), $\psi \in R^{48}$ denotes the 48 different phase settings of the 72 MW antennas in the phased array, and ℱ(⋅) is the nonlinear function approximated by our deep neural network, reflecting the physical reality that SAR distributions result from a complex interaction between the patient anatomy and tissue characteristics (encoded in ℬ) and the electromagnetic configuration of the treatment system (encoded in ψ). Our model is trained to learn this mapping directly from simulation data, enabling efficient inference of SAR distributions for new input scenarios without the need to explicitly solve Maxwell’s equations.

### 2.4. Cross-attention-based network

**Figure 3.**
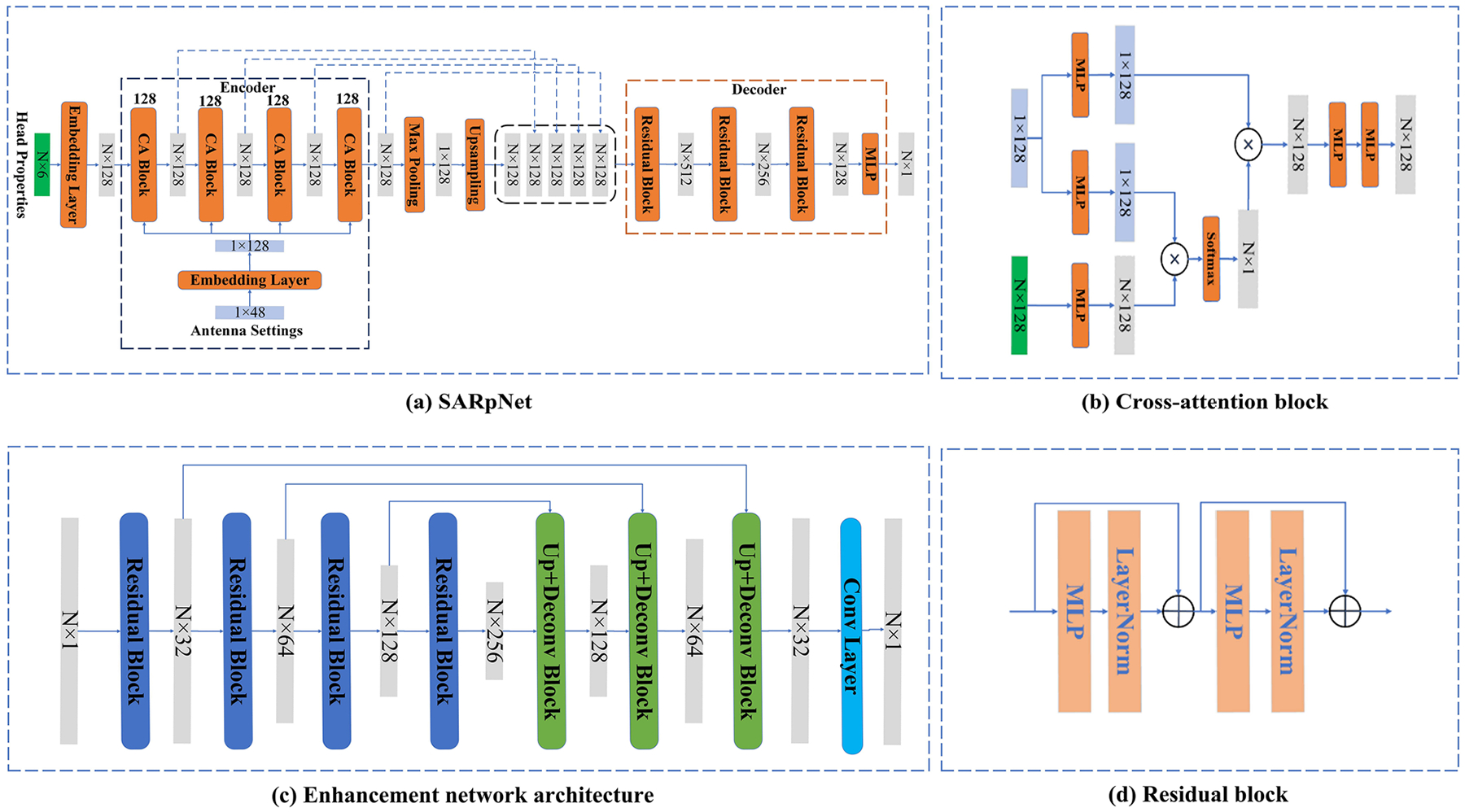
The architecture of the proposed networks in both prediction-stage and Enhance-stage. (a) Prediction network architecture. (b) Cross-attention block. (c) Enhancement network architecture (d) residual block. MLP, multi-layer perceptron; CA, cross-attention; N, sample number.

The overview of the proposed SAR prediction network (SARpNet) is shown in Figure 3(a), which is designed to learn the complex interactions between MW energy deposition, patient anatomy, and tissue characteristics. The network adopts an encoder-decoder architecture, leveraging four cross-attention blocks within the encoder to integrate antenna phases with tissue properties, which include coordinates, relative permittivity, electrical conductivity, and density. Specifically, the network takes two inputs: tissue properties represented as an N×6 array and antenna phases represented as a 48×1 array, where N is the number of 3D points in the mesh representing the brain anatomy. These inputs are passed through separate embedding layers, producing feature representations of size N×128 and 48×128, respectively.

The core innovation of SARpNet lies in the use of cross-attention modules within the encoder to dynamically model the interaction between antenna inputs and tissue properties. Conceptually, the attention mechanism operates by projecting these two input modalities into a shared latent space using learnable transformations, and computing context-aware feature representations through a similarity-based weighting scheme. The architecture of the cross-attention block is shown in Figure 3(b). To compute the attention mechanism, we project the inputs into query, key, and value vectors:

(8) $$Q=F_{a}W_{Q},K=F_{p}W_{K},V=F_{p}W_{V},$$

where $W_{Q},W_{K}$ and $W_{v}$ are learnable weight matrices. $F_{a}$ is the embedded antenna feature maps, and $F_{p}$ is the embedded brain property feature maps. Specifically, query vectors (Q) encode the energy delivery pattern driven by antenna phase configuration. Key vectors (K) capture tissue-specific absorptive properties, defining how susceptible a location is to energy accumulation. Value vectors (V) encode the spatial and electrical context of each point, used to compute SAR magnitude.

The attention map Att is derived using scaled dot-product attention:

(9) $$\text{Att}=\text{Softmax}\left(QK^{T}/\sqrt{d_{k}}\right),$$

where $d_{k}$ is the dimensionality of the query, key, and value spaces. The attention weights Att quantify the degree to which each tissue location is influenced by the global phase configuration. The attention weights Att are used to compute a weighted sum of the value vectors V:

(10) Z=AttV,

where Z is the output representation for the tissue properties conditioned on and modulated by the antenna phase interactions. To improve learning stability and refine the representation, a residual connection and a feedforward network are applied:

(11) $$H=\text{MLP}(\text{Norm}(Z))+F_{p},$$

where Norm denotes layer normalization. MLP denotes multi-layer perceptron with two fully connected layers for further feature transformations.

The first cross-attention block processes these embedded features, outputting a feature map of size N×128. In subsequent stages, the embedded antenna phases are provided to the cross-attention block, along with the output from the previous block, gradually extracting high-level features and effectively capturing the interactions between the tissue properties/anatomy and antenna settings. After the encoder, a max-pooling layer is applied to the feature map produced by the final cross-attention block to extract a global feature representation. This global feature is then concatenated with the feature maps from all preceding cross-attention blocks, creating a comprehensive local-to-global feature representation to extract multi-scale and hierarchical relationships. The local-to-global feature map is then passed to the decoder, which consists of three Residual Blocks (RBs) as shown in Figure 3(d). Each RB is built by three consistent MPLs followed by layer normalization. The RBs progressively reduce the feature size, with output dimensions of 512, 256, and 128, respectively. Finally, a single MPL is applied to predict the SAR distribution, with the size of N×128.

The SARpNet model during the prediction stage is trained using a combined loss function that includes the Structural Similarity Index Measure (SSIM) loss and L2 loss, defined as:

(12) $$L_{l}=\alpha_{1}L_{\text{ssim}}\left(P_{\text{SAR}},P_{\text{SAR}}^{*}\right)+\beta_{1}L_{2}\left(P_{\text{SAR}},P_{\text{SAR}}^{*}\right),$$

where $P_{\text{SAR}}$ and $P_{\text{SAR}}^{*}$ are the predicted SAR distribution and its corresponding ground truth, respective. $\alpha_{1}=1.0$ and $\beta_{1}=1.0$ are the training weights that have been set empirically. SSIM loss provides a measure of similarity by comparing distributions based on luminance similarity, contrast similarity, and structural similarity information. L2 loss minimizes the global differences between the predicted and ground truth SAR distributions, focusing on reducing the overall numerical discrepancies across the entire distribution. By combining L2 loss for pixel-level intensity accuracy and SSIM loss for structural similarity, the model optimizes both numerical values and spatial patterns, ensuring the SAR distribution closely aligns with the ground truth.

### 2.5. Enhancement network

To further improve prediction accuracy, we applied a 1D U-Net architecture during the enhancement-stage, as shown in Figure 3(c), to refine the prediction results from the Prediction-stage. The encoder consists of four convolutional blocks that progressively reduce the spatial dimension of the input while increasing the number of feature channels. Each block typically contains two convolutional layers followed by downsampling operations to capture high-level representations. The decoder path consists of four upsampling blocks, which progressively restore the spatial dimension while reducing the number of feature channels. Skip connections are employed to preserve high-resolution features, aiding in the reconstruction process and enhancing gradient flow during training.

Since high-intensity points (SAR values) in the target region only occupy a small portion of the total data, directly applying L2 loss can be challenging for refining these points since L2 Loss focuses on minimizing the global differences. To address this challenge, we introduce a threshold (30 W/Kg) during the training of the enhancement network. For points with intensities below this threshold, we use L1 loss to focus on fine-grained intensity refinement, while for points with intensities above the threshold, L2 loss is applied to handle larger intensity differences more effectively. This combination helps the model better handle both low and high-intensity regions, improving the overall training process and prediction accuracy.

### 2.6. Training implementation

For training, we used the Adam optimizer with an initial learning rate of 0.0001, which was reduced by a factor of 0.2 every 100 epochs, for a total of 500 training epochs. Here, one epoch refers to a complete pass through the entire training dataset during model optimization. Before training, the mesh coordinates are normalized to the range [0, 1] to ensure consistent input scaling across all training samples. The total training time was approximately 6 h. The network was implemented using the machine learning library PyTorch and run on a server equipped with a 40 GB Nvidia A100 GPU and 64 GB of RAM.

We evaluated the SAR prediction results, in terms of Root Mean Squared Error (RMSE), SSIM and Peak Signal-to-Noise Ratio (PSNR). Since the SAR distribution affects the thermal dose, we also evaluated the results by gamma index (GI), given by:

(13) $$\gamma (S)=\underset{S^{\prime }}{\text{min}}\sqrt{{\left(\frac{\Delta d\left(S,S^{\prime }\right)}{\Delta d_{\text{crit}}}\right)}^{2}+{\left(\frac{\Delta D\left(S,S^{\prime }\right)}{\Delta D_{\text{crit}}}\right)}^{2}}$$

where S and $S^{\prime }$ represents the predicted SAR and its corresponding ground truth. Δ and ΔD represents the spatial distance and dose difference, respectively. $\Delta d_{\text{crit}}$ and $\Delta D_{\text{crit}}$ represents the distance-to-agreement (DTA) criterion and the dose difference criterion, respectively. The GI results will be are reported using the commonly adopted criteria of 2%/2 mm, 3%/3 mm, and 3%/5 mm, corresponding to increasingly relaxed thresholds for dose difference and spatial agreement, respectively.

## 3. Results

The quantitative results of the prediction stage are presented in Table 2, where the model achieved an average RMSE of 5.7 W/kg, an MAE of 4.9 W/kg, and an SSIM of 0.84 for the whole brain, with a mean SAR of 7.3 W/kg. The average GIs were 83.5% (2%/2 mm), 85.7% (3%/3 mm) and 88.4% (3%/5 mm). For the target region, where the mean SAR was 31.5 W/kg, the MAE increased to 9.2 W/kg. The absolute and relative RMSE were 9.2 W/kg and 0.18, respectively, while the PSNR reached 20.7 dB. The average GIs for 2%/2 mm, 3%/3 mm and 3%/5 mm were 78.5%, 80.0% and 82.6%, respectively.

**Figure 4.**
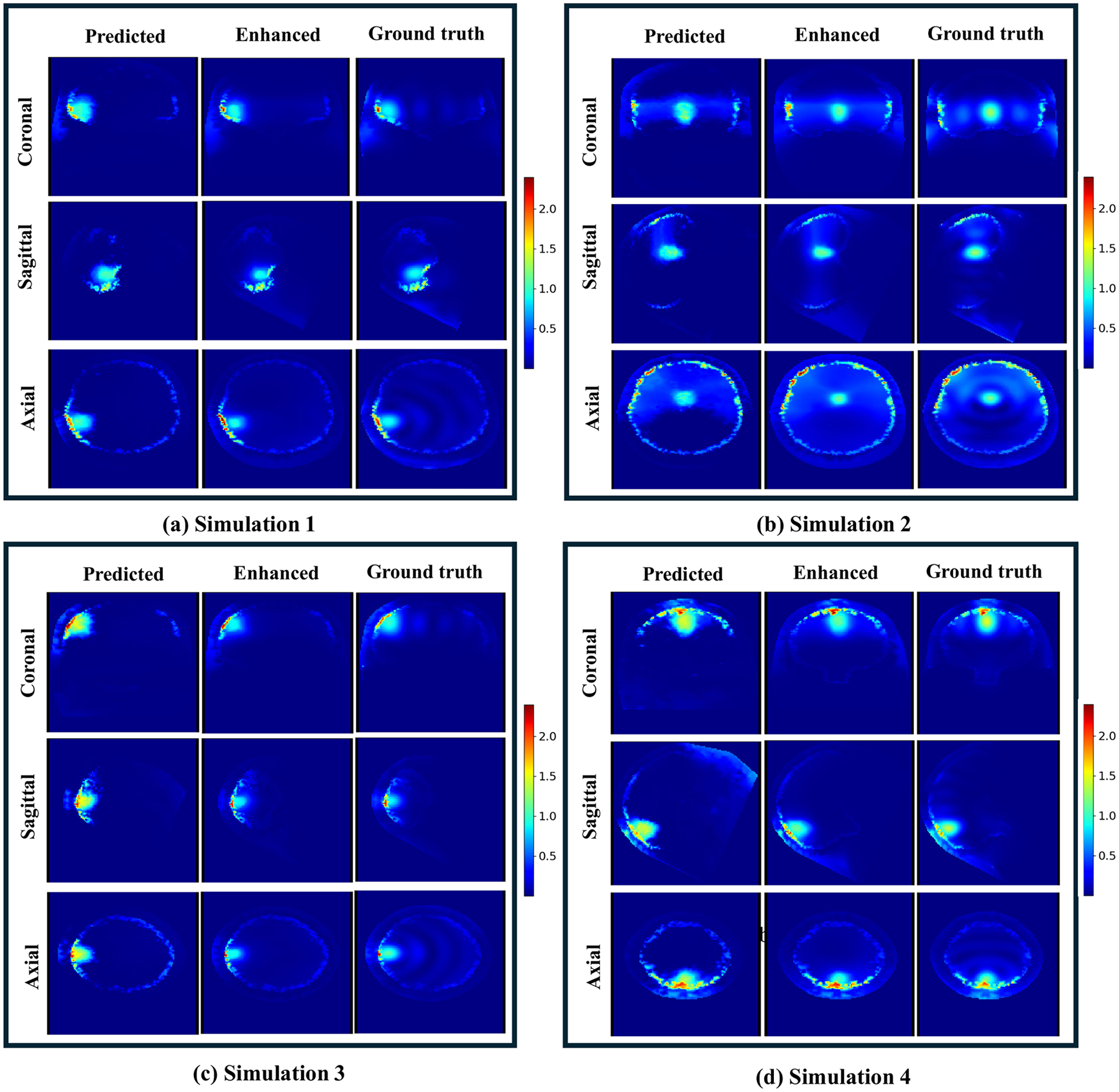
Normalized SAR prediction results of 4 example simulations out of the testing set (20 simulations). For each simulation, from top to bottom show 3 different views. From left to right shows the results from the prediction-stage, enhance-stage and the ground truth, respectively. The intensity of all images have been normalized to the maximum SAR value in the target region.

Four examples of SAR prediction results are presented in Figure 4, demonstrating that the predicted SAR distribution closely matches the ground truth, preserving the focus shape within the target region. In non-target regions characterized by the absence of significant SAR peaks, the predicted SAR remains consistently low, in agreement with the ground truth. Additionally, the peripheral regions of the brain model closest to the antennas, which received the highest SAR, also reveals good agreement with the ground truth data. This indicates that the model effectively captures key SAR distribution patterns in both target and non-target regions. Note that the SAR peaks near the skull are not expected to be of a concern since they are located within the cerebrospinal fluid (currently not included in this study) which will be cooled via forced convection induced by the temperature gradient caused by the SAR peaks [45]. Additionally, the water bolus surrounding the head circulates room-temperature water, providing further cooling to mitigate potential hotspots in superficial tissues, including those adjacent to the skull.

Compared to the prediction stage, SAR values in both the target and peripheral regions became significantly more accurate, indicating improved precision in clinically critical areas. In non-target regions without SAR peaks, the initially low SAR values left limited room for further improvement, resulting in a less noticeable enhancement effect. The quantitative results of the enhancement stage are also presented in Table 2, where the MAE and the RMSE were further reduced to 1.6 and 3.3 W/kg, and the SSIM increased to 0.90 for the whole brain, comparing with results from the prediction stage. For the target region, the MAE was reduced to 2.5 W/kg, and the absolute and relative RMSE was reduced to 4.8 W/kg and 0.05, respectively. The PSNR was improved to 26.5 dB. The average GIs after the enhancement were improved to 87.1% (2%/2 mm), 90.0% (3%/3 mm) and 91.7% (3%/5 mm) for the whole brain, and to 85.6%, 87.2% and 90.1% for the target region, respectively. These results demonstrate that our enhancement model, trained with the threshold-based loss function, effectively refines SAR values in clinically relevant regions and improves overall prediction performance.

Our approach predicts the SAR distribution for a single case in 4 s—a substantial improvement over FEM-based SAR calculations, which require an average of 10 min on a high-performance workstation with 192 GB RAM machine with a 20-core CPU (2.2–3.0 GHz).

## 4. Discussion

The findings of this study underscore the potential of deep learning-based approaches to significantly advance HTP. By leveraging a diverse and robust dataset with significant variability in tumor positions, antenna parameters, and tissue properties, our model demonstrated promising predictive capabilities for SAR distribution. The main contributions of this work are multi-fold: (1) We introduce a novel cross-attention mechanism that dynamically explores and emphasizes the interplay between antenna settings and patient-specific tissue properties and anatomy, significantly enhancing prediction accuracy. (2) By incorporating advanced deep learning techniques, our method streamlines the HTP workflow, drastically reducing the time required for FEM-based SAR estimation while minimally compromising quality, making real-time or near real-time planning feasible. To the best of our knowledge, this is the first study to develop deep learning techniques for SAR prediction, establishing a new paradigm for hyperthermia simulations.

The clinical application of this method could offer several key advantages over non-AI approaches. Most notably, the substantial reduction in computation time—from approximately 10 min to just 4 s per simulation—enables near real-time re-planning, substantially benefiting clinical decision-making. While current adaptive planning techniques that use precomputed SAR libraries per antenna [22,46] already allow near-instantaneous re-optimization, they are typically implemented in systems with lower frequency (e.g., 434 MHz [22], 70 MHz [46]) and fewer antennas (12 and 4, respectively). That being said, these adaptive methods can incorporate full inverse optimization of phase and amplitude settings, as demonstrated in Kok et al. [47], though their application has been limited to relatively low-frequency and small-array systems. In contrast, our work addresses SAR prediction at a higher operating frequency of 915 MHz using a densely populated 72-antenna phased array in the brain, a region characterized by highly convoluted anatomical structures. In this setting, FEM-based meshes may offer an advantage over finite-difference time-domain (FDTD)-based grids by providing superior geometric conformity at tissue boundaries, which can be critical for accurately modeling energy deposition in anatomically complex regions. Nonetheless, FDTD remains the most widely used method in clinical hyperthermia due to its computational efficiency, simplicity, and compatibility with voxel-based patient models. For many clinical applications, the spatial resolution achievable with FDTD is sufficient to meet treatment planning needs, as demonstrated in references [22] and [46]. Our study highlights how combining the modeling precision of FEM-based simulations with the speed of deep learning surrogates can enable both anatomical accuracy and practical usability in complex treatment scenarios such as brain hyperthermia.

Another advantage our model is the accuracy of the predicted SAR distribution, as evidenced by the low RMSE and high SSIM values and Gamma index, all indicating strong agreement with the simulation-based ground truth. The preservation of the SAR focal shape within the target region, as measured by SSIM, along with accurate SAR estimates in both target and non-target regions, suggests that the model effectively captures clinically relevant spatial patterns. The improvement achieved during the enhancement phase further underscores the model’s potential to refine initial predictions and enhance precision in critical regions. This is particularly important in hyperthermia therapy, where precise control over thermal dose is essential to maximize tumor radio-sensitization while minimizing hotspots in healthy tissue [48,49].

Despite these promising results, further investigations are warranted to fully evaluate the efficacy of the techniques here developed before implementing them for clinical use. In particular, defining clinically relevant thresholds for technical metrics such as SSIM, RMSE, and Gamma Index remains an open question, as these do not yet have established standards in HTP. Another important limitation of this study is that all simulations were performed on a single patient anatomy. Although different tissue electrical properties, tumor positions, and antenna configurations were considered, the underlying anatomy remained unchanged. This restricts the ability of the model to capture inter-patient variability, such as differences in head size, skull thickness, brain morphology, and overall tissue composition. Consequently, further validation is required using a larger number of imaging-based patient datasets that reflect a broader spectrum of patient anatomies and pathologies. In particular, explicit separation of white and gray matter, inclusion of cerebrospinal fluid, and a wider range of tumor sizes, shapes, and locations will be critical to more comprehensively assess the robustness and clinical value of this deep learning approach for 3D SAR-based HTP.

Currently, our model uses a simplified approach for phase calculation based on path-length differences. In future work, we plan to expand the model to support both phase and amplitude optimization, which is necessary to improve its flexibility and enable broader applicability across different applicator configurations and treatment scenarios. While the current work represents a significant methodological advance, its clinical translation will require substantial further development, integration, and testing within established clinical workflows. Nonetheless, this study demonstrates the feasibility and potential of our deep learning-based model as an effective approach to substantially reduce computation time in SAR prediction, addressing a key limitation of FEM-based simulations in HTP.

## 5. Conclusion

In this work, we propose a two-stage deep learning method for predicting SAR patterns as part of the HTP workflow for brain cancer treatment. The first stage, known as the Prediction stage, employs a cross-attention-based network to effectively incorporate anatomy, tissue properties, and antenna settings. The second stage known as Enhancement uses a U-net architecture to further improve prediction accuracy. Using a brain hyperthermia applicator as a case study, our method demonstrated encouraging results, achieving accurate SAR predictions with significantly reduced computation time, facilitating both pretreatment planning and near real-time HTP in anatomically complex regions. While additional validation is required, this work lays a foundation for the integration of AI-based methods into next-generation treatment planning systems for hyperthermia.

## Figures and Tables

**Table 1.** Tissue properties at 915 MHz used in the numerical SAR simulations of a five-layer human head with fat, bone, muscle, intranasal air and brain tissues obtained from the IT’IS tissue properties database v4.015.

Tissue	$\rho \left(\text{kg}/{\text{m}}^{3}\right)$	$\epsilon_{r}(-)$	σ(S/m)	λ(cm)
Air	1	1	0	32.8
DI water	1000	78.2	0.18	3.6
Fat	911	11.3	0.11	9.7
Bone	1908	12.4	0.15	9.2
Muscle	1090	55.0	0.95	4.4
White matter (W)	1041	38.8	0.60	5.2
Average of G & W	1043	45.8	0.77	4.8
Gray matter (G)	1045	52.7	0.95	4.4

The deionized (DI) water is part of the brain hyperthermia applicator. ρ: density; $\epsilon_{r}$: relative permittivity; σ: electrical conductivity; λ: wavelength.

**Table 2.** Quantitative analysis of the prediction (P) and enhancement (E) stages for the whole brain (WB) and target region (TR) using a testing speed of 2 s.

Stage	Absolute RMSE			PSNR (dB)	SSIM	GI (2%2mm)	GI (3%3mm)	GI (3%5mm)
	MAE (W/Kg)	(W/Kg)	Relative RMSE					
P (WB)	4.9 ± 0.4	5.7 ± 0.3	0.08 ± 0.02	24.9 ± 0.4	0.84 ± 0.06	83.5 ± 1.2%	85.7 ± 1.1%	88.4 ± 1.2%
P (TR)	8.1 ± 0.4	9.2 ± 0.2	0.18 ± 0.03	20.7 ± 0.6	0.75 ± 0.05	78.5 ± 1.1%	80.3 ± 1.2%	82.6 ± 1.4%
E (WB)	1.6 ± 0.2	3.3 ± 0.4	0.04 ± 0.02	29.7 ± 0.4	0.90 ± 0.05	87.1 ± 1.1%	90.0 ± 1.0%	91.7 ± 1.1%
E (TR)	2.5 ± 0.2	4.8 ± 0.3	0.05 ± 0.01	26.5 ± 0.2	0.88 ± 0.04	85.6 ± 1.3%	87.2 ± 1.1%	90.1 ± 1.1%

MAE, Mean absolute Error; RMSE, Root Mean Squared Error; PSNR, Peak signal-to-noise Ratio; SSIM, Structural Similarity Index Measure; GI, gamma Index.

## Data Availability

The data that support the findings of this study are available from the corresponding author upon reasonable request.

## Appendix. AI glossary

### Neural network:

A layered computational model inspired by the brain that learns patterns from data by adjusting connections between nodes.

#### Convolutional neural network (CNN):

A neural network architecture specialized for processing grid-like data, such as images, using convolutional layers to extract spatial features.

#### Encoder:

The part of a network that compresses input data into meaningful lower-dimensional features.

#### Decoder:

The component of a neural network that reconstructs the output (e.g., SAR distribution) from the encoded representation.

#### Cross-attention:

A mechanism that enables the model to use one input (e.g., antenna settings) to guide the interpretation of another input (e.g., tissue properties), improving contextual relevance.

#### Query, key, and value vectors:

Components of attention mechanisms where the query seeks information, the key identifies matching content, and the value provides the relevant data used in the output.

#### Transformer:

A neural architecture that uses attention mechanisms to model relationships between inputs, originally for language processing but now widely used in vision and 3D tasks.

#### Feature:

An automatically learned data representation that captures relevant patterns or structures from the input to support the prediction task

#### Perceptron:

A basic unit in a neural network that computes a weighted sum of inputs followed by an activation function.

#### Embedded map (or embedding):

A learned representation that transforms raw input data into a higher-dimensional space suitable for neural network processing.

#### U-Net:

A convolutional neural network architecture with an encoder-decoder structure and skip connections, commonly used for biomedical image segmentation.

#### Residual block:

A neural network component that adds shortcut connections to preserve information and enable training of deeper models.

#### Ground truth:

The reference standard or correct output used to evaluate model performance, typically derived from simulations or measurements.
